# Modelling the regulation of telomere length: the effects of telomerase and G-quadruplex stabilising drugs

**DOI:** 10.1007/s00285-013-0678-2

**Published:** 2013-04-26

**Authors:** Bartholomäus V. Hirt, Jonathan A. D. Wattis, Simon P. Preston

**Affiliations:** School of Mathematical Sciences, University of Nottingham, University Park, Nottingham, NG7 2RD UK

**Keywords:** Mathematical model, Telomeres, Telomerase, G-quadruplex, T-loop, RHPS4, 92B05, 37N25, 34K07

## Abstract

Telomeres are guanine-rich sequences at the end of chromosomes which shorten during each replication event and trigger cell cycle arrest and/or controlled death (apoptosis) when reaching a threshold length. The enzyme telomerase replenishes the ends of telomeres and thus prolongs the life span of cells, but also causes cellular immortalisation in human cancer. G-quadruplex (G4) stabilising drugs are a potential anticancer treatment which work by changing the molecular structure of telomeres to inhibit the activity of telomerase. We investigate the dynamics of telomere length in different conformational states, namely t-loops, G-quadruplex structures and those being elongated by telomerase. By formulating deterministic differential equation models we study the effects of various levels of both telomerase and concentrations of a G4-stabilising drug on the distribution of telomere lengths, and analyse how these effects evolve over large numbers of cell generations. As well as calculating numerical solutions, we use quasicontinuum methods to approximate the behaviour of the system over time, and predict the shape of the telomere length distribution. We find those telomerase and G4-concentrations where telomere length maintenance is successfully regulated. Excessively high levels of telomerase lead to continuous telomere lengthening, whereas large concentrations of the drug lead to progressive telomere erosion. Furthermore, our models predict a positively skewed distribution of telomere lengths, that is, telomeres accumulate over lengths shorter than the mean telomere length at equilibrium. Our model results for telomere length distributions of telomerase-positive cells in drug-free assays are in good agreement with the limited amount of experimental data available.

## Introduction

Most normal cells cycle and divide a limited number of times, a discovery first made by Hayflick (1965), who grew normal human fibroblasts in culture and observed 60–80 population doublings before apoptosis. The limited number of divisions is referred to as the Hayflick limit (Hayflick 1979). On reaching the Hayflick limit, cells cease proliferation permanently and enter a state called replicative senescence in which they are still alive and functional but do not divide. The erosion of protective structures located at the ends of chromosomes, known as telomeres, during each replication is responsible for the limited lifespan of a cell, marking the ageing of cells and eventually triggering irreversible cell cycle exit and cell death when telomeres become critically short. Unprotected telomeres are detected by the DNA repair machinery and trigger a DNA damage response characterized by the formation of telomere dysfunction-induced foci at telomeres (Takai et al. 2003). Activation of the tumour suppressor gene p53 then triggers cell cycle arrest leading to apoptotic cell death or replicative senescence through the induction of p21. Alternatively, expression of p16 can induce senescence, although this is less well understood (Deng et al. 2008).

There have been several approaches based on mathematical modelling to understand telomere length dynamics of somatic and cancerous cells and how they contribute to chromosome stability and the initiation of senescence or apoptosis. The first papers on quantitative modelling of telomere dynamics describe the process of telomere shortening by simple deterministic (Levy et al. 1992) and probabilistic (Arino et al. 1995; Olofsson and Kimmel 1999) models, which only take account of losses due to a special phenomenon called the end-replication problem causing telomeres to shorten progressively with each cycle (see Sect. 2.1). Rubelj and Vondracek (1999) extended these previously established models of telomere shortening (‘gradual telomere shortening’) by the introduction of the possibility of ‘abrupt telomere shortening’ caused by DNA repair mechanisms due to accumulation of DNA damage, producing sudden, stochastic changes in telomere length, and becoming more frequent as telomere shortening advances. Similarly, Sozou and Kirkwood (2001) and Proctor and Kirkwood (2002) included environment-dependent components effecting telomere shortening, where oxidative stress in form of endogenous reactive oxygen species produced by mutant mitochondria is assumed to be the cause of substantial telomere loss.

Telomere length is maintained in most cancer cells by the enzyme telomerase capable of adding new telomeric DNA onto chromosome ends. Mechanisms contributing to telomere length equilibrium have been considered by Blagoev (2009), who proposed a model in which telomere extension by telomerase occurs more frequently at short telomeres than at long telomeres. A logistic function describes the probability of the occurrence of an extendible state of telomeres, opposed to a capped state, which was inspired by data from experiments in Teixeira et al. (2004) on telomere elongation in yeast cells. Other work on the dynamics of telomere length by Qi (2011) compares the effects of normal ageing, accelerated ageing of patients with Werner’s syndrome and the unlimited lifespan of telomerase-positive cells, where models involve telomere-length-dependent telomere loss and gain as well as telomere-length-dependent probabilities for cell division.

Mathematical models can be a useful means for integrating different types of experimental data to predict the mechanism of action of compounds (Wolkenhauer et al. 2009). In this paper, we aim to develop and analyse differential equation models of the effects of G-quadruplex ligands on telomere length regulation in telomerase-positive cancer cells, where we consider different time-scales, from one cell cycle to a large number of cell replications.

In Sect. 2 we explain the background biology. In Sect. 3 we develop and analyse a model describing the telomere length dynamics for telomerase-positive cells during one cell cycle and investigate how they respond to treatment with the G4-stabilising drug RHPS4. This model can also be used to describe telomere length dynamics over a small number of cell generations. In Sect. 4 we investigate for what levels of telomerase and RHPS4 the telomere length distributions stabilise over a large number of cell generations, and we predict the corresponding steady-state length distributions. Section 5 summarises the results and contains a concluding discussion.

## Background biology

### Telomeres

Mammalian telomeres are specialised nucleotide sequences that protect the end of chromosomes. Telomeres contain short tandem repeats—sequences of basepairs that are repeated numerous times. The sequence of the repeat unit is TTAGGG in mammalian cells and human telomeres contain around 10–15 kilobasepairs per chromosome end at birth. Telomeres of most somatic cells typically shorten in the synthesis (S) phase of the cell cycle during each replication due to the “end-replication problem”, that is, the inability to fully replicate the terminating DNA sequences (Levy et al. 1992). One shorter and one longer telomeric end are generated during replication, where the longer strand is generally rich in guanosine (G) and devoid of cytosine (C). The single-stranded protrusion of the longer strand is referred to as the *G-overhang*, which varies between 50–500 nucleotides in mammalian cells and is considerably shorter in most other eukaryotes. About 3 basepairs are lost from one DNA strand on each round of cell division due to the end-replication problem. However, human and mouse telomeres shorten by about 50–200 basepairs during each replication at both telomeric ends and the average telomere length in human cells decreases by roughly 2–4 kilobases during their lifetime. A more likely explanation of the intensive and double-sided telomere shortening is postreplicative processing by a nuclease (Palm and de Lange 2008; Makarov et al. 1997). Also, oxidative stress in the from of reactive oxygen species, which accumulates over the lifespan of a cell, causes accidental lesion in the DNA and is assumed to be the cause of substantial telomere loss (Richter and von Zglinicki 2007; von Zglinicki et al. 2005).

This progressive telomere erosion has been designated as the reason why normal mammalian somatic cells only divide a finite number of times in vitro, before undergoing permanent growth arrest. It is, however, not yet clear whether it is the average telomere length (Martens et al. 2000) or the length of the shortest telomere (Hemann et al. 2001) that is critical for the onset of cell cycle arrest in a cell. Looking at the distribution of telomere lengths can help determine whether a percentage of short telomeres or the mean telomere length is a trigger for the onset of replicative senescence. Telomeres that become critically short, and thus unprotected, are recognised by the cell as DNA double-strand breaks, inducing senescence and apoptosis, which is dependent on the expression of the oncosuppressor gene p53. Those cells which pass this point in cell replication through inactivation of p53 continue dividing, lose all their protective telomeric DNA and enter a state called crisis, causing end-to-end joining of chromosomes and other forms of enormous genomic instability, carcinogenesis and eventually cell death (see Greenberg 2005 for a review).

### Telomerase

The enzyme telomerase can antagonise telomere shortening by association with the telomeric end, where it progressively synthesises telomeric repeat sequences at the single-stranded overhang of the telomere, thus inhibiting telomere uncapping which occurs when telomeres become too short. It has been suggested that human telomerase acts rapidly on most ($\sim$70–100 %) telomeres following replication (Wu and de Lange 2009) and requires the telomeric G-overhang for telomere elongation. In HeLa cells that were synchronised at the ${\mathrm{{G}}}_1/\mathrm{S}$ transition, the total overhang length gradually increased over the next 6–7.5 h (Zhao et al. 2009; Dai et al. 2010), indicating a phase of increased telomerase activity, and that telomeric sequences are replenished each time a cell divides. Telomerase consists of TERC (Telomerase RNA Component) with a template region for copying telomeric repeat sequences, and the catalytic protein TERT (Telomerase Reverse Transcriptase), which catalyses the G-rich extension of linear chromosomes. TERC is generally highly expressed in all cells, and independently of telomerase activity, whereas the concentration of TERT is estimated at less than 50 copies per cell. In normal somatic cells the catalytic subunit TERT is repressed, but it is upregulated in immortal cells, suggesting it is the major determinant for telomerase activity.

The majority of cancer cells express telomerase continually; they possess altered telomeres and have the potential for unlimited replication. Telomerase was found to be present in 85–90 % of cancerous cells and it is believed that its specific role is to immortalise these cells (Kim et al. 1994). Most of the remaining 10–15 % of cancer cells, in contrast, can maintain their telomeres by a telomerase-independent pathway called alternative lengthening of telomeres (ALT); see Cesare and Reddel (2010).

### Telomere structure

The extendible, open form of telomeres is presumably the most likely form occurring during telomere synthesis. Telomeres, however, can loop back and tuck their single-stranded end into the duplex DNA of telomeric sequences to form a *t-loop* (reviewed by Blackburn 2001; de Lange 2004, 2009), where a specific protein complex named shelterin (or telosome) (de Lange 2005; Palm and de Lange 2008) is involved in protecting chromosome ends from DNA degradation and DNA damage responses. The t-loop might dissolve during DNA replication; however, it is not yet known whether t-loops switch into an open state during the S phase or persist throughout the cell cycle. In our model (Sect. 3) we will assume that t-loops also function as telomerase inhibitors, as they hide the telomeric G-rich end from access by telomerase, and structural rearrangements between t-loops and the open form of telomeres allow telomerase to establish telomere length homeostasis.

Alternatively, telomeric ends can spontaneously fold into guanine-rich structures called G-quadruplexes (G4), discovered by Henderson et al. (1987), which are supported by monovalent cations such as potassium (${\mathrm{K}}^+$) in the nucleus. G4 structures form in vivo and probably unfold during telomere replication (Schaffitzel et al. 2001). When G-quadruplexes are located at the very end of the telomeric G-overhang, which has been shown to be their preferred location (Tang et al. 2008), the enzyme telomerase is inhibited by the folding of the G-rich end (Zahler et al. 1991). For reviews of G-quadruplex structures in vitro and in vivo, see Lipps and Rhodes (2009) and Knig et al. (2010). More general reviews on telomere structures and their function in chromosome-end protection can be found in Oganesian and Karlseder (2009) and Xu (2011).

Optimal telomerase activity seems to require the unfolded single-stranded form of terminal telomere sequences. Despite the length variation of individual telomeres within a cell or an organism, average telomere length is maintained within a narrow range that is specific for each species. Studies of sperm (Allsopp et al. 1992), for instance, suggest 8–20 kb in human, where germ-line cells generally express telomerase (except from mature sperm and oocytes, see Wright et al. 1996). Furthermore, data on telomere length in the telomerase-positive HeLa and MCF-7 human breast cancer cell lines (Canela et al. 2007) indicate a coefficient of variation of, respectively, 0.23 and 0.11. The stability of telomere length suggests the thesis that telomerase-positive cells establish an equilibrium between telomere attrition and elongation. However, Cristofari and Lingner (2006) found that HeLa telomeres, which were observed over 56 population doublings (PD), elongated at a constant rate of 415–635 bp/PD upon overexpression of the main functional subunits of the enzyme telomerase, the catalytic protein TERT and the telomerase RNA component (TERC). This massive telomerase activity is referred to as super-telomerase, and long telomeres did not change into a permanently non-extendible state in super-telomerase cells.

**Fig. 1:**
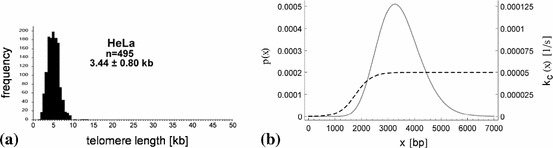
**a** A HT Q-FISH histogram of the telomere length distribution of HeLa cells, where $n=495$ nuclei were analysed, adapted from Canela et al. (2007), with permission from PNAS. **b** A gamma probability density function, $p(x)=\,\mathrm{{e}}^{-x/\theta }\,x^{\gamma -1}/(\theta ^\gamma \,\varGamma (\gamma ))$, for the telomere length in HeLa cells, with mean $L_0=3{,}440$ bp and standard deviation $\sigma _0=800$ bp, that is with the parameters $\gamma =L_0^2/\sigma _0^2$ and $\theta =\sigma _0^2/L_0$, is indicated by the solid gray line. The rate of t-loop formation (see Sect. 3 and formula (1)), $k_c(x)$, is modelled by a sigmoidal function of telomere length (*dashed line*) with shape parameters $\alpha =1{,}775$ bp, $\beta =300$ bp and $\delta =5\times 10^{-5}\, \mathrm{s}^{-1}$. Shorter telomeres are more likely to be in an unlooped form than longer telomeres

Telomere length has been measured using different techniques, among which telomere restriction fragment (TRF) analysis using Southern blotting (Kimura et al. 2010), and quantitative fluorescence in situ hybridization (Q-FISH) (Poon et al. 1999) have been frequently used. Several reviews of the techniques of telomere length measurement can be found in the literature (Saldanha et al. 2003; Dmitriev and Vassetzky 2009; Samassekou et al. 2010). A recent high-throughput (HT) Q-FISH method (Canela et al. 2007) generates telomere-length frequency histograms, and allows for the analysis of interphase nuclei. Telomere length is maintained in telomerase-positive HeLa cells, for example, with a measured mean value of $L_0=3.44$ kb and standard deviation of $\sigma _0=0.80$ kb. A HT Q-FISH histogram of the telomere length distribution of HeLa cells is shown in Fig. 1a, which we approximate by a gamma distribution in Fig. 1b, shown by a solid gray line.

Investigation of Martens et al. (2000) into normal human fibroblasts (telomerase-negative) having a limited lifespan showed that short telomeres increasingly accumulate in cells and the length distribution of telomeres becomes positively skewed close to senescence. Proctor and Kirkwood (2003) considered the uncapping of telomeres by the opening of t-loops as a trigger for replicative senescence to account for the experimental results found by Martens et al. (2000).

### Quadruplex-stabilising drugs

On the other hand, stabilisation of G-quadruplexes by specific ligands can limit telomerase activity and alter telomere function in cancer cells. Anti-cancer researchers are now trying to design G-quadruplex ligands that will mimic the effect of the metal ions and inhibit telomerase, with the aim of achieving antitumour activity through the effective stabilisation of G-quadruplexes (Monchaud and Teulade-Fichou 2008). The G-quadruplex ligands RHPS4 (3,11-difluoro-6,8,13-trimethyl-8H-quino[4,3,2-kl]acridinium methosulphate) (Heald et al. 2002) together with the 3,6,9-trisubstituted acridine compound BRACO-19 (Gunaratnam et al. 2007) and telomestatin (Tauchi et al. 2006), are promising compounds among the cancer inhibitor agents and have come close to clinical testing (Bilsland et al. 2011). These compounds all inhibit telomerase activity, limiting long term proliferation of cancer cells, and directly target components of the protective cap of telomeres, leading to immediate effects on cancer cell proliferation (Neidle 2010).

Cheng et al. (2008) compared relative quadruplex and duplex binding affinity constants of different quaternary polycyclic acridinium salts and found that quaternised quino[4,3,2-kl]-acridinium salts, such as RHPS4, selectively bind and stabilise quadruplex DNA. Also, quadruplex DNA binding affinity correlated strongly with telomerase-inhibitory activity data for these G4 ligands. Cookson et al. (2005) showed a notable reduction in telomere length of MCF-7 breast cancer cells when treated with subtoxic doses (${<}1\,\upmu \mathrm{M}$) of RHPS4. RHPS4 treatment of human melanoma lines possessing relatively long telomeres resulted in a dose-dependent decrease in cell replication and accumulation of cells in the $\mathrm{{S}}$-$\mathrm{{G}}_2/\mathrm{M}$ phase of the cell cycle (Leonetti et al. 2004). Furthermore, RHPS4 induces a marked decrease of cell growth in human cell lines such as the 21NT breast cancer cells and A431 vulval carcinoma cells after 15 days and for concentrations lower than the level of acute cytotoxicity (Gowan et al. 2001). It also rapidly induces telomere dysfunction by telomere uncapping, which leads to short-term cell death through usage of higher doses. Integrative approaches to investigate the effects of RHPS4 experimentally and by means of mathematical modelling were proposed by Johnson et al. (2011) and Hirt et al. (2012). The precise cell-cycle specific behaviour of RHPS4 and its mechanism of action in cancer cells, however, are still to be elucidated.

### Mathematical modelling

Golubev et al. (2003) used mathematical modelling to investigate possible causes for the observed positive skewness of telomere length distribution, such as DNA damage caused by free radicals. Similarly, Grasman et al. (2011) characterise the dynamics of telomere shortening by the property that longer telomeres are more vulnerable to oxidative stress, as they are larger targets. The enzyme telomerase is also active at a low level in some somatic cells (Masutomi et al. 2003), such as human fibroblasts, and telomerase-dependent shortening, leading to positively skewed telomere length distributions, has been explained by op den Buijs et al. (2004). Another mechanism yielding skewed telomere length distributions has been described by Itzkovitz et al. (2008), who developed a population mixture model with a re-populating pool of stem cells of constant telomere length and a derived pool which experiences constant decrease in telomere length, where one daughter cell of the repopulating pool stays and one transfers to the derived pool after cell replication.

Furthermore, there are a number of models of telomere-length maintenance in telomerase-positive cells. For example, Kowald (1997) developed a mathematical model involving the concentration of a capping protein, which can bind the G-overhang once it is sufficiently long, and which inhibits telomerase and facilitates DNA replication by maintenance of the single-stranded overhang it is bound to, but is released after telomere replication. To account for the assumption that the functional state of the telomere rather than its length determines the fate of a cell, Arkus (2005) considered the binding and dissociation of a telomeric protein, TRF2, to telomeric repeat sequences, assuming that TRF2 caps telomeres and inhibits telomerase. Rodriguez-Brenes and Peskin (2010) proposed another approach of modelling telomere length maintenance processes based on the biophysics of t-loop formation, which is assumed to determine the state of a telomere and also the cell’s fate. They assumed that the longer telomeres are, the more frequently telomere ends come into close proximity of internal positions of the telomere, and hence the more likely are invasions of double-stranded DNA by the G-overhang, which can be facilitated by TRF2 and results in the formation of displacement loops together with t-loops. The dynamics of t-loop formation were described by a worm-like chain model and an algorithm was developed to sample telomeric chromatin chains (modelled as semi-flexible polymer chains) at thermodynamic equilibrium. An ODE model and a stochastic model describe shortening by the end-replication problem, C-strand processing and telomerase-induced telomere elongation. In addition to telomere length maintenance, Kowald (1997) modelled the increase in telomere length when oligonucleotides are added to cell culture. On the other hand, Sidorov et al. (2003) investigated the impact of telomerase inhibition on the growth of tumours possessing either homogeneous or heterogeneous telomere length distributions.

Telomerase-independent pathways of telomere length maintenance are considered by Olofsson and Bertuch (2010), who capture the mechanisms of survivorship of individual budding yeast cells. Another approach to understanding telomere length maintenance by means of mathematical modelling is presented by Antal et al. (2007), who superimpose stochastic telomere length variations upon the systematic decrease in telomere length in ALT cells.

## Model for discrete generation numbers

**Fig. 2:**
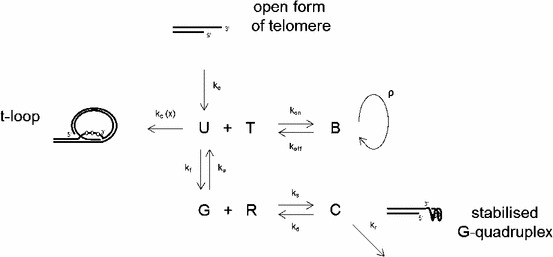
Model of telomeric states U, B, G, C. Kinetics for each reaction are described by their rate constants $k$. Free telomerase (T) and the G4-stabilising drug (R) in the nucleus bind open forms (U) and G4 structures (G), respectively. Telomerase elongation occurs at rate $\rho$. Telomeres enter the system at rate $k_e$ and exit the system due to t-loop formation at rate $k_c(x)$ and due to G4-stabilisation by a G4 ligand at rate $k_r$. Here, $x$ is the length of a telomere. The transitions $\mathrm{U}\rightarrow \mathrm{{t}}$-loop and $\mathrm{C}\rightarrow \mathrm{{stabilised}}$ G-quadruplex are assumed to be irreversible, and only through replication telomeres could leave these states

We summarise the mechanisms of telomere length regulation in a simple model containing the states U, B, G and C respectively for the number of telomeres in the open (*U*ncapped/*U*nfolded) form, those *B*ound to telomerase (T), those in a *G*-quadruplex formation and those forming a *C*omplex with the drug RHPS4 (R). The model is illustrated in Fig. 2. We refer to B, C and the t-loop in the model as the capped states, to G as the folded form of telomeres and to U as the state of telomeres that are neither capped nor folded, that is in the open form. After telomere duplication in the S phase telomeres are introduced into system at rate $k_e$ in the open form (U) state and then switch between the open and G4 forms (G4 folding rate $k_f$ and G4 unfolding rate $k_u$), where telomeres in the open form bind to free telomerase molecules $T$, with association rate $k_\mathrm{on }$ and dissociation rate $k_\mathrm{off }$, which synthesise nucleotides with rate $\rho$ at the telomere end. Telomeric intramolecular G4 structures do not allow telomerase association with the G-overhang, and can be stabilised by free G4 ligand molecules $R$, where the association and dissociation rates of RHPS4 are $k_{s}$ and $k_{d}$, respectively. We assume that one telomerase molecule binds one telomere to elongate the telomeric end, and one G4 ligand molecule is sufficient to stabilise a G4 form. Furthermore, all kinetic rates are assumed to be constant and non-negative.

We aim to simulate not only the dynamics of the average telomere length, but also of the telomere length distribution over time for control cells and cells treated with a G4-stabilising compound. We include a variable $x$ for the length of telomeres and allow for a constant influx of telomeres in the open form into the system, at rate $k_e$, and losses with rates $k_c(x)$ and $k_r$, after the formation of t-loops and locking of G4 structures by G4 ligands, respectively. We assume that the rate of t-loop formation $k_c$ is dependent on telomere length, where shorter telomeres are more likely to form t-loops than longer telomeres. In particular, we approximate the rate of the formation of t-loops by a sigmoidal function, shown as the dashed line in Fig. 1b, inspired by a quantitative model for the probability of uncapping of telomeres in mammalian cells (Proctor and Kirkwood 2003). The shorter the telomeres, the fewer binding sites for certain shelterin proteins (Griffith et al. 1999) facilitating the t-loop formation are present, and, in turn, the less likely the t-loop formation becomes. Hence the probability of uncapping is modelled as a decreasing function of telomere length. Using $x$ to denote the number of basepairs (bp) of a telomere, we model the rate of t-loop formation (telomere capping) by

1 $$\begin{aligned} k_c(x)=\frac{\delta }{1+\exp ((\alpha -x)/\beta )}, \end{aligned}$$

with shape parameters $\alpha >0$ bp, $\beta >0$ bp and $\delta >0\, \mathrm{s}^{-1}$. For small $\beta$, this has the form of a step function, with step at $x=\alpha ; k_c(x) \approx 0$ for $x<\alpha$ and $k_c(x) \approx \delta$ for $x>\alpha$; and $\beta$ describes the range of telomere lengths over which the transition occurs.

Since the average telomere loss of about $\mu =45$ bp during chromosome replication is much less than the initial telomere length of approximately 2k to 6k basepairs in HeLa cells, we treat telomere length, $x$, as a continuous variable. The dynamics of the number of individual telomeres of length $x$ at time $t$ can be mathematically described by a partial differential equation (PDE) model of the number densities of telomeres in the states U, B, G, C, that is,

2 $$\begin{aligned} \frac{\partial }{\partial t}\; U(x,t)&= k_e p(x)+ k_\mathrm{off }B(x,t)+k_{u}G(x,t) \nonumber \\&- (k_c(x)+k_\mathrm{on }T(t)+ k_{f}) U(x,t),\end{aligned}$$

3 $$\begin{aligned} \frac{\partial }{\partial t}\; B(x,t)&= k_\mathrm{on }T(t) U(x,t)- k_\mathrm{off }B(x,t)- \rho \frac{\partial }{\partial x}\; B(x,t),\end{aligned}$$

4 $$\begin{aligned} \frac{\partial }{\partial t}\; G(x,t)&= k_{f} U(x,t)+ k_{d} C(x,t)-(k_{u}+k_{s} R(t)) G(x,t),\end{aligned}$$

5 $$\begin{aligned} \frac{\partial }{\partial t}\; C(x,t)&= k_{s} R(t) G(x,t) - (k_{d}+k_{r}) C(x,t), \end{aligned}$$

where $p(x)$ is the probability density function of the length of telomeres entering the system at rate $k_e$. Assuming that telomerase and RHPS4 are conserved quantities in the system, we have

6 $$\begin{aligned} T(t)+\int _0^\infty B(x,t)\,\mathrm{{d}}x = T_0, \quad R(t)+\int _0^\infty C(x,t)\,\mathrm{{d}}x = R_0, \end{aligned}$$

for the numbers of free telomerase molecules, $T(t)$, and the numbers of free RHPS4 molecules, $R(t)$. The term $\rho \frac{\partial }{\partial x}\; B(x,t)$ is the only derivative term with respect to $x$ in the model equations and accounts for the process of telomere elongation at rate $\rho$ by telomerase.

### Steady state

We now assume that the numbers of bound telomerase and bound RHPS4 molecules are small compared respectively to the numbers of free telomerase and free RHPS4 molecules in the nucleus, that is, $T(t)\approx T_0$ and $R(t)\approx R_0$. This assumption requires $k_\mathrm{on }\ll k_\mathrm{off }$ and $k_d\gg k_s$, which will be verified later (see Table 1). Steady state telomere length distributions are described by the equations

7 $$\begin{aligned} 0&= k_e p(x) + k_\mathrm{off }B(x)+ k_{u} G(x)-( k_c(x)+k_\mathrm{on }T_0 + k_{f}) U(x),\end{aligned}$$

8 $$\begin{aligned} \rho \frac{\partial }{\partial x}\; B(x)&= k_\mathrm{on }T_0 U(x)-k_\mathrm{off }B(x),\end{aligned}$$

9 $$\begin{aligned} 0&= k_{f} U(x)+ k_{d} C(x) - (k_{u}+k_{s} R_0) G(x),\end{aligned}$$

10 $$\begin{aligned} 0&= k_{s} R_0 G(x) - (k_{d}+k_{r}) C(x), \end{aligned}$$

for each of the four telomere states $U, B, C, G$. Using Eqs. (9) and (10), we obtain $C\propto G\propto U$. We then express $U$ as a function of $B$ and $p$ using equation (7), and subsequently rewrite (8) as an ODE for the variable $B(x)$. Solving (10), (9) and (7) respectively, we find

11 $$\begin{aligned} C&= \frac{k_s R G}{k_d + k_r},\quad G = \frac{ k_f (k_r+k_d) U }{ k_u (k_d+k_r) + k_r k_s R} ,\end{aligned}$$

12 $$\begin{aligned} U&= \frac{(k_e p + k_{off} B ) [ k_u (k_d+k_r) + k_r k_s R ] }{ [ k_u (k_d+k_r)+k_r k_s R ] (k_c+ k_{on} T) + k_f k_r k_s R }, \end{aligned}$$

then the equation relating the distributions $B$ and $p$ is

13 $$\begin{aligned} \varrho B^{\prime } = - k_{off} B + \frac{ k_{on} T (k_e p + k_{off} B) [ k_u (k_d+k_r)+k_r k_s R ] }{ [ k_u (k_d+k_r)+k_r k_s R ] (k_c+ k_{on} T) + k_f k_r k_s R }, \end{aligned}$$

which has the form $p = \varLambda B + \nu B^{\prime }$ where both coefficients are $x$-dependent through the step-like $k_c(x)$.

**Table 1:** Parameter estimates for the discrete-generation model

Parameter	Description	Value
$k_\mathrm{on }$	Telomerase binding rate	$2.6\times 10^{-7}\, \mathrm{s}^{-1}$
$k_\mathrm{off }$	Telomerase dissociation rate	$2.2\times 10^{-4}\, \mathrm{s}^{-1}$
$\rho$	Rate of nucleotide addition	$6.287\times 10^{-2}\, \mathrm{{nt}}\,\mathrm{s}^{-1}$
$k_{f}$	G4 folding rate	$1.6\times 10^{-2}\, \mathrm{s}^{-1}$
$k_{u}$	G4 unfolding rate	$3.8\times 10^{-3}\, \mathrm{s}^{-1}$
$K$	RHPS4 equilibrium binding constant	$1.4\times 10^{-5}$
$k_{s}$	RHPS4 binding rate	$10^{-7}~\mathrm{s}^{-1}$
$k_{d}$	RHPS4 dissociation rate	$7.1\times 10^{-3}~\mathrm{s}^{-1}$
$k_e$	Telomere influx rate	$1.5\times 10^{-2}\, \mathrm{s}^{-1}$
$k_r$	Telomere loss rate	$5\times 10^{-6}\, \mathrm{s}^{-1}$
$\delta$	Maximum formation rate of t-loops	$5\times 10^{-5}~\mathrm{s}^{-1}$
$\alpha$	Parameter describing the value $x$ at which $k_c(x)=\frac{\delta }{2}$	1,775 bp
$\beta$	Parameter describing the range of $x$ over which the transition $k_c(x)$ occurs	300 bp
$L_0$	Mean length of HeLa telomeres	3,440 bp
$\sigma _0$	Standard deviation of HeLa telomere length	800 bp
$\mu$	Average telomere loss per cell replication	45 bp

### Gamma-distributed input

If we temporarily restrict ourselves to consider the lengths $x>\alpha$ and assume $\beta \ll \alpha$ so that $k_c=\delta$, then we can find explicit forms for the distribution. We write ${\widehat{x}} = x- \alpha$ so we are only concerned with ${\widehat{x}}\ge 0$. The form $p(\,{\widehat{x}}\,) = \varLambda B(\,{\widehat{x}}\,) + \nu B^{\prime }(\,{\widehat{x}}\,)$ has special solutions in terms of the Gamma distribution. We model the input function $p(\,{\widehat{x}}\,)$ as a gamma distribution with parameters $\gamma$ and $\theta$, hence

14 $$\begin{aligned} p(\,{\widehat{x}}\,)=\frac{1}{\varGamma (\gamma )\theta ^\gamma } \;{\widehat{x}}^{\gamma -1}\,\mathrm{{e}}^{-\frac{{\widehat{x}}}{\theta }}. \end{aligned}$$

Choosing the parameters such that $\gamma =L^2/\sigma ^2$ and $\theta =\sigma ^2/L$ we obtain a distribution with mean telomere length $L$ and variance $\sigma ^2$. Choosing the parameters $\theta =\nu /\varLambda$, we find that both $B(\,{\widehat{x}}\,)$ and $p(\,{\widehat{x}}\,)$ are Gamma-distributed with

15 $$\begin{aligned} B({\widehat{x}}) = \frac{ {\widehat{x}}^{\gamma -1} \mathrm{e}^{-\varLambda {\widehat{x}}/\nu } \varLambda ^{\gamma -1}}{\varGamma (\gamma ) \nu ^\gamma },\quad \mathrm{{and}} \quad p(\,{\widehat{x}}\,) = \frac{ {\widehat{x}}^{\gamma -2} \mathrm{e}^{-\varLambda {\widehat{x}}/\nu } \varLambda ^{\gamma -1}}{\varGamma (\gamma -1) \nu ^{\gamma -1}}. \end{aligned}$$

Hence, the effect of the process is to increase the exponent from the input value ($p$) of $\gamma -1$ to the output ($B$) value $\gamma$. Since the Gamma distribution has mean $\gamma \theta$, and variance $\gamma \theta ^2$, the overall effect of the process illustrated in Fig. 2 is to increase the mean from the input value of $(\gamma -1) \nu /\varLambda$, being the mean of the input function $p(\,{\widehat{x}}\,)$, to $\gamma \nu /\varLambda$, as the mean of $B$, the output. The standard deviation is also increased by the process. These solutions satisfy the boundary conditions $p,B\rightarrow 0$ as ${\widehat{x}}\rightarrow 0^+,\infty$.

### Gaussian-distributed input

We now return to the more general case, where $x<\alpha$ is permitted, and impose the boundary condition $B(-\infty )=0$ or $B(+\infty )=0$ on (8). This boundary condition is chosen such that to avoid negativity in $B(x)$ (imposing $B(0)=0$ leads to a sign change of $B(x)$ in $x=0$ due to $p(x)>0$ for all $x\in \mathbb{R }$).

In order to investigate which parameters control the shape of the telomere length distributions at steady state, we derive approximate analytical expressions for the distributions. Assuming $\gamma =L^2/\sigma ^2$ is sufficiently large and using the central limit theorem, we approximate the gamma distribution $p$ with the Gaussian distribution

16 $$\begin{aligned} {\tilde{p}}(x)=\frac{1}{\sqrt{2\pi \sigma ^2}} \exp \left( -\frac{(x-L)^2}{2\sigma ^2}\right) , \end{aligned}$$

having the same mean, $L$, and variance, $\sigma ^2$, as $p$. We also assume $\beta$ is small and so approximate $k_c(x)$ in (1) by $\delta \,\mathrm{{H}}(x-\alpha )$, where $\,\mathrm{{H}}(x)$ denotes the Heaviside step function, to derive (approximate) analytical expressions at steady state for $U(x), B(x), G(x), C(x)$, and the mean telomere length of telomeres leaving the system, $k_c(x)U(x) + k_rC(x)$. We use an integrating factor to solve the ODE (8) with the boundary conditions $B(-\infty )=0$ and $B(+\infty )=0$, to obtain

17 $$\begin{aligned} B(x)&= \left\{ \begin{array}{ll} a_1e^{-a_2x} \left( 1 + \mathrm {erf}\left( \frac{x-L}{\sigma \sqrt{2}}- \frac{\sigma }{\sqrt{2}} a_2\right) \right) , &{}\quad x<\alpha ,\\ b_1e^{-b_2x} \left( b_0 + \mathrm {erf}\left( \frac{x-L}{\sigma \sqrt{2}}- \frac{\sigma }{\sqrt{2}} b_2\right) \right) , &{}\quad x>\alpha , \end{array} \right. \end{aligned}$$

18 $$\begin{aligned} U(x)&= \left\{ \begin{array}{ll} \frac{1}{c_1} (k_\mathrm{off }B(x) + k_e{\tilde{p}}(x)), &{}\quad x<\alpha ,\\ \frac{1}{\delta + c_1} (k_\mathrm{off }B(x) + k_e{\tilde{p}}(x)), &{}\quad x>\alpha , \end{array} \right. \end{aligned}$$

19 $$\begin{aligned} G(x)&= \frac{k_f(k_d+k_r)}{k_uk_d+k_r(k_u+k_sR_0)}U(x), \end{aligned}$$

20 $$\begin{aligned} C(x)&= \frac{k_sR_0}{k_d+k_r}G(x), \end{aligned}$$

where

21 $$\begin{aligned} a_2 = \frac{k_\mathrm{off }}{\rho }\left( 1-\frac{k_\mathrm{on }T_0}{c_1}\right) , \quad b_2 = \frac{k_\mathrm{off }}{\rho }\left( 1-\frac{k_\mathrm{on }T_0}{\delta + c_1}\right) , \end{aligned}$$

and

22 $$\begin{aligned} c_1 = k_\mathrm{on }T_0 + \frac{k_f k_s k_r R_0}{k_u k_d+k_r(k_u+k_s R_0)}, \end{aligned}$$

and $a_1, b_0, b_1$ are explicit expressions involving kinetic parameters of the system that are too complex to display here; for details see Hirt (2012). All constants satisfy $a_i, b_i>0$. Here, the steady state distribution of $B$ is a product of an exponential function and an error function, where the error function dominates the exponential function for $x<\alpha$ and *vice versa* for $x>\alpha$. The parameter $b_2$ determines how rapidly the telomere length distribution approaches zero for increasing telomere lengths, $x$, with smaller values of $b_2$ increasing the positive skewness of the distribution. Hence, decreasing the rate of t-loop formation (by lowering $\delta$) increases the positive skewness of $B(x)$, and so does increasing the number $T_0$ of telomerase molecules (or the rate, $\rho$, of telomere elongation), for example.

The parameters $k_\mathrm{off }$ and $k_e$ function as scaling factors that determine the contribution of $B(x)$ and ${\tilde{p}}(x)$ to the telomere length distribution $U(x)$. For small $k_\mathrm{off }$, when $B(x)$ is increasingly positively skewed, we expect $B(x)$ to have a larger tail than $U(x)$ due to the respectively decreasing and increasing contributions of $B(x)$ and ${\tilde{p}}(x)$ to $U(x)$. We note that $U(x)$ is independent of the rates $k_f$ and $k_u$ of G4 folding and unfolding, respectively, for control cells ($R_0=0$). The distributions of $G$ and $C$ are of the same shape as $U(x)$, where larger $R_0$ increases the ratio of telomere numbers $C/G$. The ratio of telomere numbers $C/U$ increases with increasing $R_0$ in a nonlinear fashion, and tends to $k_f/k_r$ for large $R_0$. On the other hand, the ratio of telomere numbers $G/U$ decreases with increasing $R_0$ in an inverse fashion, and is equal to $k_f/k_u$ for $R_0=0$. For larger $T_0$, the distributions become increasingly positively skewed.

By integrating the Eqs. (7)–(10) over the interval $[-\infty ,\infty )$, assuming $B(-\infty )=B(\infty )=0$, and taking the sum of all these equations, we obtain the steady-state input-output balance

23 $$\begin{aligned} k_e = \int _{-\infty }^\infty k_c(x)U(x)~\,\mathrm{{d}} x + k_r\int _{-\infty }^\infty C(x)~\,\mathrm{{d}}x, \end{aligned}$$

where $k_c(x)$ is given by (1). We have confirmed that this holds for the solutions plotted later.

An analytic expression for the mean telomere length, $\widehat{L}=\widehat{L}(T_0,R_0)$, of telomeres leaving the system at steady state, is

24 $$\begin{aligned} \widehat{L}&= \frac{\int _{-\infty }^\infty x k_c(x)U(x) + x k_r C(x)\, \,\mathrm{{d}} x}{\int _{-\infty }^\infty k_c(x)U(x) + k_r C(x)\, \,\mathrm{{d}} x} \nonumber \\&= \frac{1}{k_e}\int _{-\infty }^\infty x k_c(x)U(x)\, \,\mathrm{{d}} x + \frac{k_r}{k_e}\int _{-\infty }^\infty x C(x)\, \,\mathrm{{d}} x, \end{aligned}$$

and an approximate formula for (24), based on the approximation of $k_c(x)$ by $\delta \,\mathrm{{H}}(x-\alpha )$, has been derived using MATHEMATICA with a series of variable substitutions and simplifications, as the formulae involved in the computation are long and complex. We consider only the limiting case $R_0 = 0$ (no drug) for $\widehat{L}$, whence

25 $$\begin{aligned} \widehat{L} = L + \frac{\rho }{2k_\mathrm{off }}\;\mathrm{erfc}\left( \frac{L-\alpha }{\sigma \sqrt{2}}\right) + \frac{k_\mathrm{on }T_0 \rho }{k_\mathrm{off }\delta }, \end{aligned}$$

where $\,\mathrm{{erfc}}(x)=1-\,\mathrm{{erf}}(x)$ is the complementary error function, and we assume positive concentrations of telomerase, $T_0>0$, and $L>\alpha$. Whereas the last term in (25) is dependent on telomerase, and mean telomere length increases linearly with the concentration of telomerase, the second term in (25) is small (${\approx }0.63$ nt) relative to the final term (${\approx }37$ nt for $T_0=25$), when we adopt the parameter values in Table 1 and choose $L=L_0-\mu$. This telomerase-independent term is due to short telomeres, which can stay in the system for an extremely long time, lengthening slowly only leaving when $x>\alpha$. Approximating $k_c(x)$ by a step function gives rise to the discontinuous limit of $\widehat{L}$ as $T_0\rightarrow 0$, but $\widehat{L}=L$ when $T_0=0$.

The expression $\widehat{L}$ is independent of $k_e$, and in the limiting case $R_0=0, \widehat{L}$ is also independent of $k_f, k_u, k_s, k_d$ and $k_r$. An increase in the parameter values $T_0$ (or $\rho$) leads to a linear increase in (25), becoming nonlinear for positive values of $R_0$. An increase in $\sigma$ leads to an increase in $\widehat{L}$ for $R_0=0$.

### Numerical solutions

We aim to show numerical results of telomere length distributions over a few generations. To derive steady state distributions at the end of each replication, we initially assume the length distribution of telomeres before the first replication event to be Gaussian $p_0(x)$ as in (16) with $L=L_0$ and $\sigma =\sigma _0$. Telomeres shorten at an average amount $\mu$ due to the end-replication problem and postreplicative processing, and consequently enter the system with length distribution $p(x)=p_0(x+\mu )$. By numerically integrating (8) and using (23) we simulate the telomere length probability density function of telomeres leaving the system, $p_1(x)=(k_c(x)U(x)+k_r C(x))/k_e$, at steady state and compare it to the distribution $p_0(x)$ of telomeres entering the system before telomere shortening takes place. Assuming telomere length does not change between the ${{\mathrm{{S/G}}}}_2$ phases of subsequent cell divisions, we treat the output telomere lengths of one cycle as the input telomere length for the next generation, $p=p_1(x+\mu )$. In particular, we use the steady state distribution of telomeres leaving the system at the end of the initial generation $i=0$ as input (of rate $k_e$) into the system at the beginning of generation $i=1$ and after telomere loss of amount $\mu$ occurred, that is $(k_c(x+\mu )U(x+\mu )+ k_r C(x+\mu ))/k_e=p_1(x+\mu )$ replacing $p(x)$ in Eq. (2), and derive steady state distributions $p_i(x)$ for higher generations $i$ in this fashion.

In general, there is only very little data on the rate parameters available in the literature. We estimate $k_e=1.5\times 10^{-2}\, \mathrm{s}^{-1}$ according to an approximate influx of $2\times 2\times 82$ telomeres [after telomere duplication and considering HeLa cells with a modal chromosome number of 82, as determined by Macville et al. (1999)] into the system per 6 h, that is the time during which telomere extension occurs (Zhao et al. 2009), and estimate, within biologically feasible ranges, the values $\alpha , \beta$ and $\delta$ for the sigmoid function $k_c(x)$ (compare Fig. 1b) and the loss rate $k_r$, as shown in Table 1. In order to adopt appropriate values for the remaining parameters, given in Appendix, we employ the fact that a concentration of 1 nM corresponds to approximately $10^{-9} N_A V_n\,\mathrm{{mol}}\,\mathrm{l}^{-1} \approx 415$ molecules per HeLa nucleus, where $N_A=6.022\times 10^{23}~\mathrm{{mol}}^{-1}$ is the Avogadro constant and $V_n=6.9\times 10^{-13}$ l is the nuclear volume of a HeLa cell. The BioNumbers data base of Milo et al. (2010) provides us with this average volume for HeLa nuclei, which is taken from Monier et al. (2000). Using standard conversion factors, we obtain the rate parameters shown in Table 1. Note that $k_\mathrm{on }\ll k_\mathrm{off }$ and $k_d\gg k_s$ as required by the earlier assumption that $T(t)\approx T_0$ and $R(t)\approx R_0$ for the numbers of bound telomerase and RHPS4 molecules, respectively. The exact values for each of the rates of RHPS4 binding, $k_{s}$, and RHPS4 dissociation, $k_d$, are not relevant for the steady state of the system, so we set $k_{s}=10^{-7}~\mathrm{s}^{-1}$ (following the size of the other molecular binding kinetic parameter, $k_\mathrm{on }$), for example, and derive $k_d=k_s K^{-1}$ from the RHPS4 equilibrium binding constant, $K$.

**Fig. 3:**
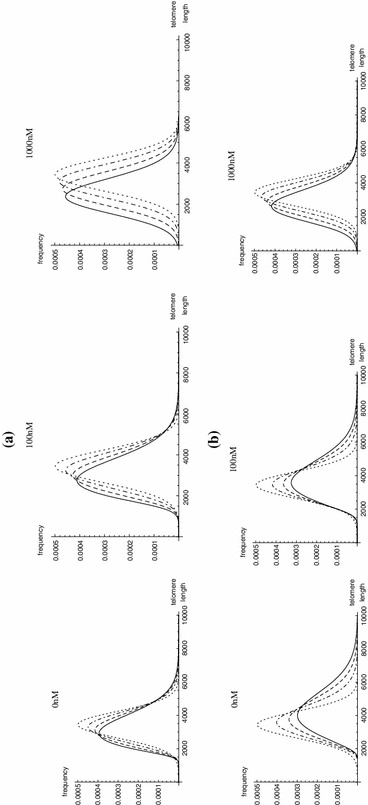
Simulations of length probability density distributions $p_i(x)$, for telomeres entering the open system at generations $i=0,10,20,30$ (*dotted*, *dot-dashed*, *dashed*, *solid line*) for **a** $T_0=25$ and **b** $T_0=50$, and different concentrations of RHPS4 (0, 100 and 1,000 nM). The initial mean telomere length ($i=0$) is assumed to be $L_0=3{,}440$ bp and telomeres shorten by $\mu =45$ nt each replication. The $x$-axis represents telomere length in units of basepairs, and the mean telomere lengths at generation $i=30$ are 3,352 bp (4,439 bp), 3,114 bp (3,997 bp) and 2,475 bp (2,808 bp) at $T_0=25$ ($T_0=50$) and for 0, 100 and 1,000 nM of RHPS4, respectively

Figure 3 shows simulations of telomere length distributions, $p_i(x)$, of telomeres entering the system at generations $i=0,10,20,30$, for different concentrations of RHPS4 for the cases $T_0=25$ (telomere length equilibrium) and $T_0=50$ (super-telomerase cells), respectively. For increasing drug concentrations, $R_0$, the telomere length distributions are shifted towards zero and become less positively skewed. The value $\rho =6.287\times 10^{-2}\, \mathrm{nt}\,\mathrm{s}^{-1}$ has been chosen in the simulations, as the telomere length distribution for telomeres leaving the system for $T_0=25$ is in good agreement with experimental data from HeLa cells (see Sect. 4.2).

### Summary

We developed and analysed a model of telomere length dynamics for a single division event, which describes the length regulation by telomerase and a G4-stabilising drug. We solved the model numerically and iterated the replication process over few generations. We now investigate the long term evolution over many generations.

## Model with continuous time

**Fig. 4:**
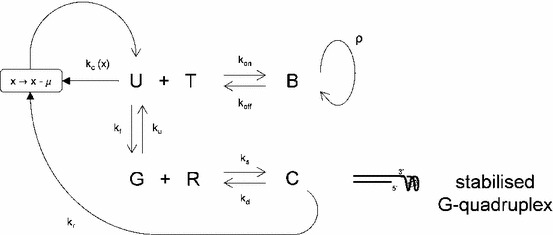
A closed model of telomeric states U, B, G, C, with telomeres of length $x$ losing $\mu$ basepairs when they exit the system by t-loop formation (rate $k_c(x)$) or after forming a complex with RHPS4 (rate $k_r$); telomeres re-enter the system in the open (U) form. Kinetics for each reaction are described by their rate constants $k$. Free telomerase (T) and RHPS4 (R) in the nucleus bind open telomere forms (U) and G4 structures (G), respectively. Telomerase elongation occurs at rate $\rho$ in the bound state, B

In order to investigate changes in the telomere length distribution over a large number of cell divisions, we now modify the model (2)–(5). We feed the telomeres that exit the system with rates $k_c(x)$ and $k_r$ back into the system in the unfolded form, assuming that telomeres shorten by an amount $\mu$ due to the end replication problem (in the S phase) before they re-enter the system. The strategy we employed to simulate telomere length distributions over several generations worked well for smaller generation numbers $i$, but is computationally too expensive for large values of $i$. Figure 4 illustrates a modified version of the model (2)–(5), allowing for analysis of the telomere length distribution after large numbers of cell divisions, that is iterative ${\mathrm{{S/G}}}_2$ phases, where we assume that telomere length does not change in the $\mathrm{{G}}_0/\mathrm{{G}}_1$ and the M phase of the cell cycle.

We aim to analyse the model dynamics and compare the output telomere lengths $k_c(x)U(x)+k_r C(x)$ to experimental telomere length measurements. We replace $k_e p(x)$ in (2) by $k_c(x+\mu )U(x+\mu ,t)+k_r C(x+\mu ,t)$ and obtain the resulting closed system of differential equations of (3)–(5) together with

26 $$\begin{aligned} \frac{\partial }{\partial t} U(x,t)&= k_c(x+\mu )U(x+\mu ,t)+k_r C(x+\mu ,t)+k_\mathrm{off }B(x,t)+k_{u}G(x,t)\nonumber \\&- (k_c(x)+k_\mathrm{on }T(t)+k_{f})U(x,t), \end{aligned}$$

which is a partial differential-delay equation.

We are interested in analysing the system’s steady-state telomere length distributions and predicting how telomerase and/or RHPS4 affect telomere length distributions over large numbers of cell divisions.

### Steady states

Assuming that steady state solutions exist is a major assumption, the validity of which we must consider carefully; for example, there may not be enough telomerase to maintain a steady state, or there may be too much telomerase and telomere length may increase without limit. Hence we expect a window in parameter space of feasible steady solutions. We assume that $T(t)\approx T_0$ and $R(t)\approx R_0$ hold at steady state and hence obtain the approximate steady state equations

27 $$\begin{aligned} 0&= k_c(x+\mu )U(x+\mu )+k_r C(x+\mu ) + k_\mathrm{off }B(x) + k_{u}G(x) \nonumber \\&- (k_c(x) + k_\mathrm{on }\, T_0 + k_{f}) U(x),\end{aligned}$$

28 $$\begin{aligned} \rho \frac{\partial }{\partial x}\; B(x)&= k_\mathrm{on }T_0 U(x) - k_\mathrm{off }B(x),\end{aligned}$$

29 $$\begin{aligned} 0&= k_{f}U(x) + k_{d} C(x) - (k_{u}+k_{s}R_0)G(x),\end{aligned}$$

30 $$\begin{aligned} 0&= k_{s}R_0 G(x) - (k_{d}+k_{r}) C(x). \end{aligned}$$

Solving (27), (29), (30) for $B$ as a function of $U(x)$ and $U(x+\mu )$, we obtain

31 $$\begin{aligned} B(x) = \frac{1}{k_\mathrm{off }} ((g(x)+k_\mathrm{on }T_0) U(x) - g(x+\mu )U(x+\mu )), \end{aligned}$$

with

32 $$\begin{aligned} g(x) = k_c(x) + c_R, \end{aligned}$$

where

33 $$\begin{aligned} c_R = \frac{k_f k_s k_r R_0}{k_u k_d+k_r(k_u+k_s R_0)}. \end{aligned}$$

Inserting (31) into the ODE (28) reduces the governing equations to a single delay differential equation in $U(x)$, namely

34 $$\begin{aligned} 0&= \frac{\rho }{k_\mathrm{off }}(g(x)\!+\!k_\mathrm{on }T_0)U^{\prime }(x) - \frac{\rho }{k_\mathrm{off }} g(x\!+\!\mu )U^{\prime }(x+\mu )\!+\!\left( g(x)+\frac{\rho }{k_\mathrm{off }}g^{\prime }(x) \right) U(x) \nonumber \\&- \left( g(x+\mu )+\frac{\rho }{k_\mathrm{off }}\,g^{\prime }(x+\mu )\right) U(x+\mu ). \end{aligned}$$

Now we aim to find approximate solutions to Eq. (34) by using quasi-continuum approximations. Such approximations have previously been used for nonlinear waves in advance-delay equations (Collins 1981; Rosenau 1986; Wattis 1996).

By defining $y=x+\mu /2$, we rewrite (34) as

35 $$\begin{aligned} 0&= \frac{\rho }{k_\mathrm{off }}((gU)^{\prime } + k_\mathrm{on }T_0U^{\prime }) \left( y-\frac{1}{2}\mu \right) - \frac{\rho }{k_\mathrm{off }}(gU)^{\prime }\left( y+\frac{1}{2}\mu \right) +(gU)\left( y-\frac{1}{2}\mu \right) \nonumber \\&-(gU)\left( y+\frac{1}{2}\mu \right) . \end{aligned}$$

It is useful to introduce the notation $\partial _y$ for the differential operator $\partial /\partial y$, and use $\partial _y^{n}$ to denote $\partial ^n/\partial y^n$. For analytic functions, $f(y)$, and $\alpha \in \mathbb{R }$, we express

36 $$\begin{aligned} f(y+\alpha ) = \,\mathrm{{e}}^{\alpha \partial _y} f(y), \end{aligned}$$

where $\,\mathrm{{exp}}(\alpha \partial _y) = \sum _{n=0}^\infty \alpha ^n\partial _y^{n}/n!$ is a differential operator which yields the Taylor series at $y$, that is,

37 $$\begin{aligned} f(y+\alpha ) = \,\mathrm{{e}}^{\alpha \partial _y} f(y) = f(y) + \alpha \,f^{\prime }(y) + \frac{\alpha ^2}{2!}f^{\prime \prime }(y) + \frac{\alpha ^3}{3!}f^{\prime \prime \prime }(y)+\cdots , \end{aligned}$$

when applied to a function $f$.

Assuming $U$ is analytic, we use (36) with $\alpha =\mu /2$ and $\alpha =-\mu /2$ and re-formulate (35) as

38 $$\begin{aligned} \,\mathrm{{e}}^{-\frac{1}{2}\mu \partial _y} \left( \frac{k_\mathrm{on }T_0\rho }{k_\mathrm{off }}\partial _y \right) U + \left( \,\mathrm{{e}}^{-\frac{1}{2}\mu \partial _y} - \,\mathrm{{e}}^{\frac{1}{2}\mu \partial _y} \right) \left( \frac{\rho }{k_\mathrm{off }}\partial _y + 1 \right) (gU)= 0. \end{aligned}$$

Rearranging using commutativity of operator multiplication yields

39 $$\begin{aligned} gU&= -\left( \,\mathrm{{e}}^{-\frac{1}{2}\mu \partial _y} - \,\mathrm{{e}}^{\frac{1}{2}\mu \partial _y} \right) ^{-1} \left( \frac{\rho }{k_\mathrm{off }}\,\partial _y + 1\right) ^{-1} \left( \,\mathrm{{e}}^{-\frac{1}{2}\mu \partial _y}\right) \left( \frac{k_\mathrm{on }T_0\rho }{k_\mathrm{off }}\partial _y \right) U \nonumber \\&= \mathfrak{A }U, \end{aligned}$$

where $\mathfrak{A }$ is an operator. Expanding the components of $\mathfrak{A }$ to third order in $\mu$, we find

40 $$\begin{aligned} \exp \left( -\frac{1}{2}\mu \partial _y\right) = 1-\frac{1}{2}\mu \partial _y + \frac{\mu ^2}{8}\partial _y^2 - \frac{\mu ^3}{16}\partial _y^3 + \mathcal{O }(\mu ^4), \end{aligned}$$

and

41 $$\begin{aligned} \exp \left( -\frac{1}{2}\mu \partial _y\right) - \exp \left( \frac{1}{2}\mu \partial _y\right)&= - \mu \partial _y - \frac{\mu ^3}{24}\partial _y^3 + \mathcal{O }(\mu ^5)\nonumber \\&= (-\mu \partial _y)\left( 1+\frac{\mu ^2}{24}\partial _y^2 + \mathcal{O }(\mu ^4)\right) , \end{aligned}$$

and for the inverse operators

42 $$\begin{aligned} \left( 1+\frac{\mu ^2}{24}\partial _y^2\right) ^{-1}&= 1-\frac{\mu ^2}{24}\partial _y^2 + \mathcal{O }(\mu ^4), \end{aligned}$$

and

43 $$\begin{aligned} \left( \frac{\rho }{k_\mathrm{off }}\partial _y +1 \right) ^{-1} = 1-\frac{\rho }{k_\mathrm{off }}\partial _y + \frac{\rho ^2}{k_\mathrm{off }^2}\partial _y^2 - \frac{\rho ^3}{k_\mathrm{off }^3}\partial _y^3 + \mathcal{O }\left( \frac{\rho ^4}{k_\mathrm{off }^4}\right) , \end{aligned}$$

where we write $f(x)=\mathcal{O }(h(x))$ if there exists a constant $M$ such that $\vert f(x)\vert < Mh(x)$ for sufficiently large $x$. Hence, assuming the quantities $\mu$ and $\rho /k_\mathrm{off }$ are small (from Table 1, $\mu = 45$ nt, $\rho /k_\mathrm{off }\approx 286$ nt) compared to $x\approx L_0=3{,}440$ nt, from (39) we obtain

44 $$\begin{aligned} gU=\frac{k_\mathrm{on }T_0\rho }{\mu k_\mathrm{off }} \left[ 1 - \left( \frac{1}{2}\mu +\frac{\rho }{k_\mathrm{off }}\right) \partial _y \right] U + \mathcal{O }( \mu ^2). \end{aligned}$$

Note that the integration constant that appears when one applies $\partial _y^{-1}$ to $U$ is equal to zero, as we assume $U^{(j)}(x)\rightarrow 0$ as $x\rightarrow \pm \infty$ for $j=0,1,2,3$. Thus we obtain the first-order differential equation

45 $$\begin{aligned} \left( \frac{1}{2}\mu +\frac{\rho }{k_\mathrm{off }}\right) \frac{\,\mathrm{{d}}U}{\,\mathrm{{d}}y} = \left( 1 - \frac{\mu k_\mathrm{off }g(y)}{k_\mathrm{on }T_0 \rho }\right) U, \end{aligned}$$

which approximately describes the telomere length distribution $U(x)$ at steady state.

We rewrite (45) as

46 $$\begin{aligned} c_0c_T\frac{\,\mathrm{{d}}U}{\,\mathrm{{d}}x} +(g(x)-c_T)U = 0, \end{aligned}$$

by defining

47 $$\begin{aligned} c_0 = \frac{1}{2}\mu +\frac{\rho }{k_\mathrm{off }},\quad c_T = \frac{k_\mathrm{on }T_0\rho }{\mu k_\mathrm{off }}, \end{aligned}$$

and use (46) to analyse how different numbers $T_0$ and $R_0$ affect the distribution of telomeres in the system at steady state. Equation (46) is a separable ODE, which we solve by integrating with respect to $x$ and using (32) and (1) for $g(x)$ and $k_c(x)$, respectively, thus

48 $$\begin{aligned} \,\mathrm{{ln}}U(x) = - \frac{\beta \delta }{c_0c_T}\,\mathrm{{ln}} \left( \,\mathrm{{e}}^{x/\beta }+\,\mathrm{{e}}^{\alpha /\beta }\right) + \frac{1}{c_0}\left( 1-\frac{c_R}{c_T}\right) x + s_0, \end{aligned}$$

where $s_0\in \mathbb{R }$ is a constant that depends on the initial number of telomeres in the system. We simplify (48) to obtain

49 $$\begin{aligned} U(x) = Ae^{\lambda x}\,\mathrm{{sech}}^{\frac{\beta \delta }{c_0c_T}} \left( \frac{x-\alpha }{2\beta } \right) , \end{aligned}$$

where

50 $$\begin{aligned} A = \exp \left( -\frac{\delta (2\beta \ln 2 + \alpha )}{2c_0c_T}\right) \exp (s_0), \end{aligned}$$

and

51 $$\begin{aligned} \lambda = \frac{2c_T-2c_R-\delta }{2c_0c_T}. \end{aligned}$$

The amplitude of the distribution is determined by $A$. For example, we may choose $s_0$ and hence $A$, such that $\int _{-\infty }^{\infty } U(x)\,\,\mathrm{{d}} x = 1$, that is, $U(x)$ is a probability density function. The quantity $\lambda$ describes the skewness of the distribution, $\,\mathrm{{sech}}$ being a symmetric bell-like curve.

### Parameter range of steady state solutions

Having constructed a steady-state approximation for the solution (49), and noted at the start of Sect. 4.1 that presuming the existence of a steady state is a significant assumption, we now analyse the conditions under which such a solution may be expected to be relevant. Necessary conditions for a steady solution, $U(x)$, of the continuum model (27)–(30) to be a distribution are that $U$ must have a maximum at a point ${\widehat{x}}$ where $U^{\prime }(\,{\widehat{x}}\,)=0$ and $U(x)\ge 0$ for all $x\in \mathbb{R }$. From (46) it follows that $g(\,{\widehat{x}}\,)=c_T$ must be satisfied for the relevant values of $T_0\ge 0$ and $R_0\ge 0$ in order for $U$ to be physical. We note that solutions $U(x)>0$ for $x<0$, representing a positive probability of telomeres with a negative length, should be regarded as unphysical. The probability of telomeres with length $x<0$, however, is usually very small in our simulations and we interpret the occurrence of larger proportions of telomeres with negative length to reflect the presence of telomeres that have lost all their telomeric sequences and are no longer functional. Such telomeres would typically cause a cell to become senescent or undergo apoptosis. In the following, we aim to find conditions on $T_0$ and $R_0$ that must be satisfied to yield solutions with $U\gneqq 0$, and we require ${\widehat{x}}>0$ for such solutions to be physical.

It follows immediately from the definitions (32) and (1) that $g(x)<\delta + c_R$ for all $x\in \mathbb{R }$, and hence by (46) and (47) $c_T<g<\delta + c_R$ in $x>{\widehat{x}}$, providing us with an upper bound for the number $T_0$ of telomerase molecules, from (33) and (47)

52 $$\begin{aligned} T_0 < T_\mathrm{max }(R_0)= \frac{\mu k_\mathrm{off }}{k_\mathrm{on }\rho } \left( \delta + \frac{k_f k_s k_r R_0}{k_u k_d+k_r(k_u+k_s R_0)} \right) . \end{aligned}$$

For high concentrations of RHPS4 namely as $R_0\rightarrow \infty$ we find $T_\mathrm{max }(R_0)\rightarrow T^\infty _\mathrm{max }= \mu k_\mathrm{off }\,(\delta + k_f)/(\rho k_\mathrm{on })$.

By the same reasoning as for (52) and since, from (32), $g(x)>c_R$ for all $x\in \mathbb{R }$, we find $c_T>c_R$, which provides us with a lower bound on $T_0$, namely

53 $$\begin{aligned} T_0 > T_\mathrm{min }(R_0)= \frac{\mu k_\mathrm{off }}{k_\mathrm{on }\rho } \frac{k_f k_s k_r R_0}{k_u k_d+k_r(k_u+k_s R_0)}. \end{aligned}$$

Thus, for high concentrations of RHPS4 ($R_0\rightarrow \infty$) we find $T_\mathrm{min }(R_0)\rightarrow T^\infty _\mathrm{min }= \mu k_\mathrm{off }k_f/(\rho k_\mathrm{on })$. For physical solutions $U(x), {\widehat{x}}$ needs to be positive, and since $g$ is monotonic increasing in $x$, we need $g(\,{\widehat{x}}\,)> g(0) = c_R + \delta /(1+\,\mathrm{{e}}^{\alpha /\beta })$, which provides a larger lower bound on $T_0$ than (53), namely

54 $$\begin{aligned} T_0 > \tilde{T}_\mathrm{min }(R_0)&= T_\mathrm{min }(R_0)+ \frac{\mu k_\mathrm{off }\delta }{k_\mathrm{on }\rho (1+\,\mathrm{{e}}^{\alpha /\beta })}\nonumber \\&= \frac{\mu k_\mathrm{off }}{k_\mathrm{on }\rho } \left( \frac{k_fk_sk_rR_0}{k_uk_d+k_r(k_u+k_sR_0)} + \frac{\delta }{1+\,\mathrm{{e}}^{\alpha /\beta }} \right) . \end{aligned}$$

Note that in the limit $\beta \rightarrow 0\, \tilde{T}_\mathrm{min }=T_\mathrm{min }$, however, in the figures below we use $\beta =300$ bp.

For $\tilde{T}_\mathrm{min }<T_0<T_\mathrm{max }$ we expect steady state solutions, for $T_0<\tilde{T}_\mathrm{min }$ the amount of telomerase is insufficient and the telomere length decays causing the cell to become senescent or undergo apoptosis. For $T_0>T_\mathrm{max }$ telomere length grows without limit.

Alternatively, we reformulate these inequalities to provide a lower and an upper bound on $R_0$, for given $T_0>0$, in a similar way to (52) and (53), that is,

55 $$\begin{aligned} R_0 > R_\mathrm{min }(T_0) = \frac{k_u(k_\mathrm{on }\rho T_0 - \mu k_\mathrm{off }\delta )(k_d+k_r)}{k_sk_r[ \mu k_\mathrm{off }(k_f + \delta )-k_\mathrm{on }\rho T_0]}, \end{aligned}$$

and

56 $$\begin{aligned} R_0 < R_\mathrm{max }(T_0) = \frac{k_\mathrm{on }\rho T_0 k_u(k_d+k_r)}{k_s k_r (\mu k_\mathrm{off }k_f - k_\mathrm{on }\rho T_0)}, \end{aligned}$$

for $\mu k_\mathrm{off }k_f>k_\mathrm{on }\rho T_0$ ($T_0<T^\infty _\mathrm{min }$), respectively. Note that $R_\mathrm{max }(T_0) \rightarrow \infty$ as $T_0 \rightarrow (T^\infty _\mathrm{min })^{-}$ and there is no upper bound on $R_0$ for $T_0>T^\infty _\mathrm{min }$. Similarly, Eq. (55) is valid for $T_0<T^\infty _\mathrm{max }$ only (where $R_\mathrm{min }(T_0)>0$) with $R_\mathrm{min }(T_0) \rightarrow \infty$ as $T_0 \rightarrow (T^\infty _\mathrm{max })^{-}$, and no physical solutions $U(x)$ exist for $T_0\ge T^\infty _\mathrm{max }$, as $T^\infty _\mathrm{max }> T_\mathrm{max }(R_0)$ (note $k_f<c_R$) for all $R_0 \ge 0$. If $\delta >k_f$, there is a range of telomerase concentrations, $T^\infty _\mathrm{min }< T_0 < T_\mathrm{max }(0)=\mu k_\mathrm{off }\delta /(\rho k_\mathrm{on })$, where no upper bound on $R_0$ exists for solutions $U(x)$ to be physical, and this is not true for $\delta <k_f$, because then $T_\mathrm{max }(0) < T^\infty _\mathrm{min }$.

**Fig. 5:**
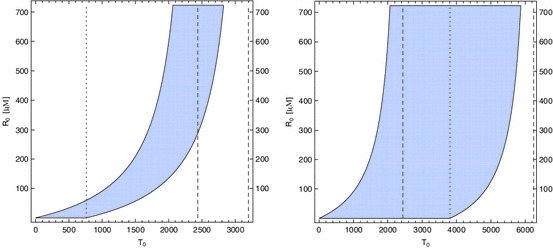
$T_0$-$R_0$-region of physical steady state solutions $U$ for $\delta = 5\times 10^{-3}\, \mathrm{s}^{-1}$ ($\delta < k_f$, *left plot*) and $\delta = 2.5\times 10^{-2}\, \mathrm{s}^{-1}$ ($\delta >k_f$, *right plot*). The rate of telomerase-induced telomere synthesis is $\rho = 2.5\times 10^{-1}~\mathrm{{nt}}\,\mathrm{s}^{-1}$. The lower ($T_\mathrm{min }(R_0)$) and upper ($T_\mathrm{max }(R_0)$) bounds on $T_0$ are defined by (53) and (52), respectively, and there is no visible difference between the lower bound $T_\mathrm{min }(R_0)$ and the larger lower bound $\tilde{T}_\mathrm{min }(R_0)$, defined by (54). *The dotted line* indicates the upper bound on telomerase, $T_\mathrm{max }(0)$, for the case of no drug and the *two dashed lines* in each plot indicate the lower ($T^\infty _\mathrm{min }$) and upper ($T^\infty _\mathrm{max }$) bounds on telomerase for large concentrations of RHPS4, where the values of $\tilde{T}^\infty _\mathrm{min }$ could not be distinguished from the values of $T^\infty _\mathrm{min }$ in these plots and are not shown

The lower and upper bounds on $R_0$ or $T_0$ can be used to plot ($T_0,R_0$)-regions of parameter space where steady physical solutions $U(x)$ exist, we can then identify the effects of changes of telomerase and RHPS4 concentrations in the cell. Examples with different values of $\delta$ are given in Fig. 5 to illustrate the cases $\delta <k_f$ and $\delta >k_f$, where we choose $\rho = 2.5\times 10^{-1}\,\mathrm{{nt}}\,\mathrm{s}^{-1}$ to illustrate the shape of these regions (lower values of $\rho$ result in unphysically large values of $T^\infty _\mathrm{min }$, for example $T^\infty _\mathrm{min }>10^5$ for $\rho =4.5\times 10^{-3}\, \mathrm{{nt}}\,\mathrm{s}^{-1}$). For $\delta > k_f$, there exists a range of telomerase concentrations where a steady state solution exists no matter how large or how small the concentration of RHPS4 is. For $\delta < k_f$, the region of steady state solutions is much smaller, hence more care for the regulation of telomerase and/or RHPS4 is required. We believe this latter case to be the more physically relevant by our choice of parameter values in Table 1, where $\delta \ll k_f$.

**Fig. 6:**
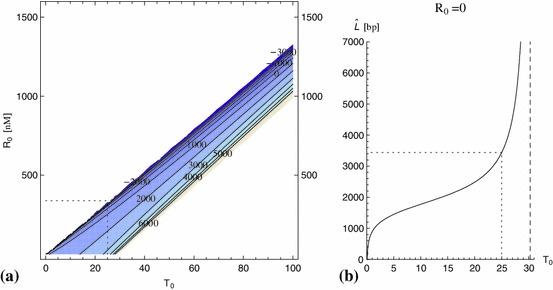
**a** Contour plot of the mean telomere length $\widehat{L}$ in $(T_0,R_0)$ space for $\rho = 6.287\times 10^{-2}\, \mathrm{{nt}}\,\mathrm{s}^{-1}$ and $\delta = 5\times 10^{-5}~\mathrm{s}^{-1}$. *The dotted lines* indicates the best approximation for $T_0$ such that $\widehat{L} = L_0$ when $R_0=0$ ($T_0=25$), and the according upper limit $R_\mathrm{max }(T_0)$. **b** A plot of the mean telomere length $\widehat{L}$ of telomeres $k_c(x)U(x)+k_rC(x)$ exiting the system per unit time as a function of the number $T_0$ of telomerase molecules for the case of no drug ($R_0=0$) in the system ($\rho =6.287\times 10^{-2}\, \mathrm{{nt}}\,\mathrm{s}^{-1}, \delta = 5\times 10^{-5}\, \mathrm{s}^{-1}$). The dotted lines indicate the value $L_0$ and according value $T_0$

For smaller (and more realistic) values of $\rho$, one is likely to find simpler $T_0$-$R_0$-regions in the form of a band in the $T_0$-$R_0$-plane as illustrated in Fig. 6. The mean telomere length of telomeres leaving the system at steady state has been computed using the same formula as for ${\widehat{L}}$ in (24) and tends to $-\infty$ for large values of $R_0$ and small values of $T_0$, and to $+\infty$ for small values of $R_0$ and large values of $T_0$. Figure 6 shows a contour plot of $\widehat{L}$ as a function of $T_0$ and $R_0$ for $\rho =6.287\times 10^{-2}\, \mathrm{{nt}}\,\mathrm{s}^{-1}$ (see below for explanation), and a plot of $\widehat{L}$ as a function of $T_0$ for $R_0=0$.

**Fig. 7:**
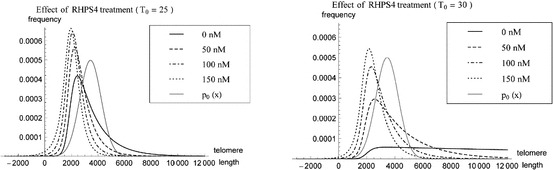
Simulations of telomere length probability density distribution $\widehat{p}(x)$ at steady state of the system (3)–(5), (26) per unit time for varying concentrations of RHPS4 (*solid line* $R_0 = 0$ nM, *dashed line* $R_0 = 50$ nM, *dot-dashed line* $R_0 = 100$ nM, *dotted line* $R_0 = 150$ nM) and the probability density distribution of the input telomere lengths, $p_0(x)$ (*solid gray line*, see Sect. 3.4 for more details). In all cases $\rho =6.287\times 10^{-2}\, \mathrm{{nt}}\,\mathrm{s}^{-1}, \delta =5\times 10^{-5}~\mathrm{s}^{-1}$ and $T_0=25$ (*left plot*) or $T_0=30$ (*right plot*). The $x$-axis represents telomere length in units of basepairs

We simulate the approximate telomere length distributions of telomeres leaving the system at steady state. The bounds on $T_0$ for physical solutions $U(x)$ at $R_0=0$, that is

57 $$\begin{aligned} \tilde{T}_\mathrm{min }(0) = \frac{\mu k_\mathrm{off }\delta }{k_\mathrm{on }\rho (1+ \,\mathrm{{e}}^{\alpha /\beta })} < T_0 < T_\mathrm{max }(0), \end{aligned}$$

are $1\le T_0 \le 30$ for $\rho =6.287\times 10^{-2}\, \mathrm{{nt}}\,\mathrm{s}^{-1}$. Figure 7 shows two plots of telomere length distributions with $T_0=25$ and $T_0=30$ and varying concentrations of $R_0$. Whereas an increase of $T_0$ leads to more skewed telomere length distributions, an increase in $R_0$ causes telomere length distributions to become less skewed. In Table 2 we give the mean telomere length $\widehat{L}$ with their respective standard deviations $\widehat{\sigma }$, which are computed using the probability density function

58 $$\begin{aligned} \widehat{p}(x) = \frac{ k_c(x)U(x) + k_r C(x)}{\int _{-\infty }^\infty k_c(x)U(x) + k_rC(x)\, \,\mathrm{{d}} x}. \end{aligned}$$

**Table 2:** Mean telomere lengths, $\widehat{L}$, with their respective standard deviations, $\widehat{\sigma }$, of telomeres leaving the system at steady state. Data are shown for the continuous-time model and two different values of telomerase molecule numbers, $T_0$

$T_0$	RHPS4 (nM)	$\widehat{L}$ (nt)	$\widehat{\sigma }$ (nt)
25	0	3,440	1,517
	50	2,706	966
	100	2,303	789
	150	1,975	768
30	0	12,201	6,549
	50	4,265	2,363
	100	3,017	1,304
	150	2,477	988

**Fig. 8:**
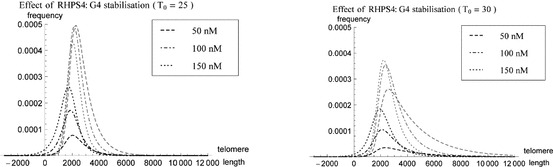
Proportions of the telomere length distribution $\widehat{p}(x)$ for telomeres $k_c(x)U(x)$ (*gray lines*) and $k_rC(x)$ (*black lines*) and varying concentrations of RHPS4 (*dashed line* $R_0 = 50$ nM, *dot-dashed line* $R_0 = 100$ nM, *dotted line* $R_0 = 150$ nM). In all cases $\rho =6.287\times 10^{-2}\, \mathrm{{nt}}\,\mathrm{s}^{-1}, \delta =5\times 10^{-5}~\mathrm{s}^{-1}$ and $T_0=25$ (*left plot*) or $T_0=30$ (*right plot*). The $x$-axis represents telomere length in units of basepairs

**Fig. 9:**
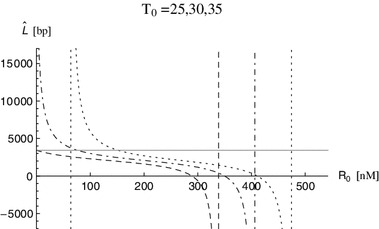
A plot of the mean telomere length $\widehat{L}$ of telomeres $k_c(x)U(x)+k_rC(x)$ exiting the system per unit time as a function of the concentration $R_0$ of RHPS4 for three different numbers of telomerase molecules, $T_0=25,30,35$, represented by the *dashed*, *dot-dashed* and *dotted line*, respectively. *The solid, gray line* indicates the value $L_0$

The decreasing positive skewness with increasing concentrations of RHPS4, $R_0$ shown in Fig. 7 for $T_0=25$ and $T_0=30$ is predominantly caused by large numbers of telomeres forming a complex with RHPS4, whose length is overall shorter than the length of telomeres leaving the system when they are in the open form, as illustrated in Fig. 8. The dependence of the mean telomere length $\widehat{L}$ on the concentration of RHPS4, $R_0$, is shown in Fig. 9 for chosen values of $T_0=25,30,35$.

The value $\rho =6.287\times 10^{-2}\, \mathrm{{nt}}\,\mathrm{s}^{-1}$ for the lengthening of telomeres by telomerase has been chosen in the simulations, as the telomere length distribution for telomeres leaving the system for $T_0=25$ is in good agreement ($\widehat{L} = 3{,}440\pm 1{,}517$ nt) with experimental data from HeLa cells (compare Figs. 1a, 7). The choice of a smaller value of $\rho$ would yield much larger values for the number of telomerase molecules, that is $T_0 \approx 400$ for $\bar{\rho } = 4.5\times 10^{-3}\, \mathrm{{nt}}\,\mathrm{s}^{-1}$ (mean value from experimental measurements, see Appendix) at telomere length equilibrium. Slightly smaller values of $\rho$ with corresponding larger values of $T_0$ can produce telomere length distributions being similar to each other. Having more knowledge of the values $\rho$, therefore, will help us determine the number of telomerase molecules in the nucleus more accurately. The small value of $T_0=25$ is, however, consistent with the average value of about 20–50 telomerase molecules per HEK-293 (human embryonic kidney) nuclei measured by Cohen et al. (2007), which is the only quantitative measurement of telomerase levels in cells we are aware of in the literature.

## Discussion and conclusions

We have investigated the effects of G-quadruplex interactive agents, such as RHPS4, on telomere erosion in telomerase-positive cells, and developed mathematical models of telomere length dynamics. In particular, we considered telomere length dynamics during the $\mathrm{{S/G}}_2$ phases, when telomerase replenishes telomeric sequences of open t-loops but not the G-quadruplex structure at each cell division. We determined steady-state length distributions, over small and large numbers of cell generations, with and without treatment by RHPS4.

We derived approximate analytic expressions, and simulated numerically, steady-states of the length distributions of telomeres, as cancer cells exit the cell cycle. We analysed the effects of different levels of telomerase and various concentrations of RHPS4 on telomere length during the $\mathrm{{S/G}}_2$ phases and considered how these effects evolve over large numbers of generations. Our models predict a positively skewed length distribution of telomeres and are in good agreement with experimental observations of HeLa cells. Moreover, our predicted value of the number of telomerase molecules in the nucleus is consistent with experimental findings in cancer cells.

In our models we assumed that t-loops and G4 structures are the key inhibitors of telomerase, and telomerase-induced telomere elongation. Constant telomere shortening due to the end-replication problem and C-strand resection, described by a constant loss term, $\mu$, in our model for a population of continuously cycling cells, that is (3)–(5) and (26), complete the mechanisms that determine the shape of telomere length distributions. We modelled telomere elongation as a length-dependent process in that shorter telomeres are more likely to remain in an open state and be extendible by telomerase than longer telomeres, as longer telomeres have a higher tendency to coil up and form t-loops.

We further assumed that telomeres only exit the system by t-loop formation, unless G4 structures are locked by RHPS4 binding, when telomeres leave the $\mathrm{{G}}_2$ phase of the cell cycle in the complex state and may unfold either at a later stage or at the beginning of the next cell cycle. We also supposed that the concentrations of telomerase and RHPS4 do not change over cell generations.

We have estimated most of the kinetic parameters in each system by using experimental results from the literature, and estimated the remaining parameters in order to reproduce the experimental results of HeLa cells obtained by Canela et al. (2007). Our model results agree well with the experimental telomere length distribution of HeLa cells and suggests a low concentration of about $T_0 = 25$ telomerase molecules per nucleus. In the literature the telomerase processivity parameter $\rho$ is given in the range $1.2-7.7\times 10^{-3}\, \mathrm{{nt}}\,\,\mathrm{{s}}^{-1}$. We investigated the sensitivity of the results to $\rho$ within this range. For small $\rho$ we obtained narrower distributions of telomere lengths (and larger values of $T_0$) than in experimental data. We therefore fitted $\rho$ using a larger value, $\rho = 6.287\times 10^{-2}\, \mathrm{{nt}}\,\mathrm{s}^{-1}$, to describe the experimental data. There is almost no visible change in the telomere length distribution for smaller numbers of $T_0$ ($\le \!100$) during one $\mathrm{{S/G}}_2$ phase as simulated by the model for a population of telomeres undergoing a single replication event, described by Eqs. (2)–(5), but visible changes occur after 5–30 generations, as shown in Fig. 3 with $T_0=25$ and $T_0=50$.

Our simulations of conditions on $T_0$ and $R_0$ for physically relevant steady solutions showed that the range of values $T_0\ge 25$ reproduces well the experimental results in the literature, and telomeres grow unboundedly in length for values of $T_0$ larger than 30. In contrast, telomeres shorten below physical lengths (or indefinitely) if we use drug concentrations larger than ${\sim }100$ nM of RHPS4 (see Fig. 7). Hence, the equilibrium state is rather sensitive to the amount of telomerase and RHPS4 in the system: larger doses of RHPS4 lead to continuous telomere shortening, whilst telomerase overexpression, on the other hand, induces continuous telomere lengthening. The steady telomere length increase we found for large $T_0$ is consistent with the findings of Cristofari and Lingner (2006), who observed elongation of telomeres at a constant rate in super-telomerase HeLa cells for over 50 population doublings. Hence, telomere length homeostasis cannot be established with telomerase overexpression.

Our model suggests two different effects of the treatment with RHPS4 which are dependent on the drug concentration used: low concentrations reduce telomere length, but do not impair the ability of the system to maintain an equilibrium, and high concentrations destabilise the system leading to chromosome degradation and senescence and/or cell death. Additionally, overexpression of telomerase can counteract telomere degradation; however, telomerase addition should be carefully regulated to maintain the system in equilibrium and not trigger unlimited telomere elongation.

Note that the upper limit for $R_0$, when telomere equilibrium can still be maintained, is probably lower than we predicted. The threshold value for telomere length as determined by the Hayflick limit (Hayflick 1979), triggering senescense or apoptosis pathways in a cell terminating cell proliferation, is likely to be based on the shortest telomere in the cell, not the average length (Hemann et al. 2001; Abdallah et al. 2009). As far as we are aware, telomere length distributions for cells exposed to different drug concentrations of RHPS4 have not yet been measured experimentally. Measurements of telomere length distributions of telomerase-positive cells at different population doublings and for varying concentrations of the drug RHPS4 or different concentrations of telomerase will be useful in comparing our model predictions to experimental results.

We found that higher concentrations of telomerase can lead to ongoing telomere lengthening, which is consistent with observations from experiments with telomerase-positive cells. We derived regions of different telomerase and RHPS4 levels that provide physically plausible solutions to our model of telomere length dynamics over large numbers of generations and showed that telomerase expression must be strictly regulated for telomere length maintenance. Too high concentrations of RHPS4 can lead to progressive telomere erosion; we estimate that drug concentrations larger than $\approx$100 nM impair the equilibrium of the system leading to continuous telomere shortening and triggering senescence and apoptosis.

In summary, the main results of this paper are showing how telomerase acts as a regulator for telomere length, and how RHPS4 can disrupt this regulation as illustrated by Figs. 6b and 9. At small concentrations, RHPS4 has little effect, but there is a critical concentration above which telomerase is unable to maintain a steady state, and rapid shortening occurs. The region of telomerase-RHPS4 parameter space where steady states exist has also been determined and is illustrated in Fig. 5.

Promising directions for future work include modelling other players involved in telomere maintenance. For instance, the shelterin protein POT1 is involved in several processes at the telomere end, which might be an interesting avenue to follow in future work. For example, two distinct functions of the protein have been identified, depending on the position of POT1 at the 3’-overhang: if POT1 is bound at the very end of the overhang (leaving less than 8 nt free at the 3’ end), telomerase cannot extend the telomere (Lei et al. 2005). On the other hand, the formation of G-quadruplex structures require that POT1 is not bound to the terminal four telomeric sequences involved in G-quadruplex formation. Since G-quadruplexes form spontaneously at the end of telomeres and are in a dynamic equilibrium with unfolded or partially unfolded forms, POT1 binding of unfolded structures may trap telomeres in the open form (Zaug et al. 2005). It may be interesting in future studies to analyse the effects of different levels of POT1 binding, in particular POT1 being overexpressed or suppressed in telomerase-positive cells.

## Appendix: Estimation of kinetic model parameters

Some experimental results on the kinetic rate and equilibrium binding constants which we use in model simulation and analysis can be found in the literature. HeLa cells are telomerase-positive cells which were taken from a cervical cancer patient in 1951. The HeLa cell line is immortal, but when telomerase activity is inhibited in HeLa nuclei, telomeres shorten by ${\sim }45$ bp/cell division (Zhao et al. 2009).

Binding kinetics of telomerase to single-stranded telomeric $\mathrm{{(TTAGGG)}}_3$ sequences (no G4 folding possible) have been measured at $37\,^{\circ }$C in vitro. Wallweber et al. (2003) determine the dissociation rate of telomerase as $k_\mathrm{off }=0.013\, \mathrm{{min}}^{-1}$, which is equivalent to a half-life of $t_{1/2}=\,\mathrm{{ln }}2/k_\mathrm{off }\approx 53$ min of the complex, and the equilibrium dissociation (Michaelis–Menten) constant has been measured at $K_m\approx 2$ nM. We estimate the binding rate of telomerase as $k_\mathrm{on }=k_\mathrm{off }/K_m=6.5\times 10^{-3}\, \mathrm{{min}}^{-1}\mathrm{{nM}}^{-1}$.

The typical number ($R_{1/2}$) of telomeric repeats synthesised before half-life $t_{1/2}$ of the telomerase-telomere complex has been estimated by Wang et al. (2007) to be between 0.66 and 4.1, that is, between about 4 and 25 bases, depending on the level of POT1-TPP1 complexes used in assays. We derive the rate of telomere elongation by $\rho = R_{1/2}/t_{1/2}$, hence we find $\rho \in [1.2\times 10^{-3},7.7\times 10^{-3}]$ in units of $\mathrm{{nt}}\,\mathrm{s}^{-1}$ with mean value $\bar{\rho }=4.5\times 10^{-3}\,\mathrm{{nt}}\,\mathrm{s}^{-1}$.

Zhao et al. (2004) studied the formation of telomeric quadruplex structures by measuring the folding ($k_f$) and unfolding ($k_u$) rate constants of the human telomere sequence $\mathrm{{(TTAGGG)}}_4$ as 150 mM of $\mathrm{K}^+$ (typical intracellular concentration) at $37\,^{\circ }\mathrm{C}$. The rate constants were determined as $k_f=1.6\times 10^{-2}\, \mathrm{s}^{-1}$ and $k_u=3.8\times 10^{-3}\, \mathrm{s}^{-1}$, hence G4 structures and the unfolded, single-stranded form exist in vitro in equilibrium with equilibration occurring slowly, with half lives of about 3 min and less than 1 min, respectively.

The equilibrium binding constant of RHPS4 with quadruplex forming human telomeric sequences $\mathrm{d}[\,\mathrm{{AG}}_3\, (\,\mathrm{{TTAGGG}})_3]$ has been estimated by Cheng et al. (2008) as $K=2.70\times 10^5\, \mathrm{M}^{-1}$ and $K=110.0\times 10^5\, \mathrm{M}^{-1}$ depending on the experimental methods used. We estimate $\bar{K}$ by choosing the mean of these values, $K=5.6\times 10^6\mathrm{M}^{-1}$.
